# Development and Validation of a Rapid High-Performance Liquid Chromatography Method for Simultaneous Determination of Methylxanthines and Flavanols in Cocoa Husk Tea

**DOI:** 10.3390/molecules31101697

**Published:** 2026-05-17

**Authors:** Thanarat Boonchalaem, Prapas Tienprateep, Kongsak Boonyapranai

**Affiliations:** 1 School of Health Sciences Research, Research Institute for Health Sciences, Chiang Mai University, Chiang Mai 50200, Thailand; thanarat_boon@cmu.ac.th; 2 Bara Scientific Co., Ltd., Bangkok 10500, Thailand; prapath@barascientific.com; 3 Research Institute for Health Sciences, Chiang Mai University, Chiang Mai 50200, Thailand; 4 Clinical Research Center for Food and Herbal Product Trials and Development (CR-FAH), Faculty of Medicine, Chiang Mai University, Chiang Mai 50200, Thailand

**Keywords:** cocoa husk tea, methylxanthine, flavanols, theobromine, HPLC–PDA, method validation

## Abstract

Cocoa husk tea has gained attention as a value-added beverage from cocoa processing by-products, due to its potential content of bioactive compounds associated with health benefits. However, rapid and reliable analytical methods for the simultaneous determination of methylxanthines and flavanols in this matrix remain limited. This study aimed to develop and validate a rapid HPLC–PDA method for the simultaneous determination of methylxanthines and selected flavanols in cocoa husk tea. Separation was performed using a Zorbax 300SB-C18 with a gradient system of acetic acid, water and acetonitrile, and detection at 280 nm. The method enabled separation of theobromine, caffeine, catechin, epicatechin, procyanidin B1, and procyanidin B2 within 15 min. Validation followed ICH Q2(R2) guidelines, demonstrating satisfactory linearity, sensitivity, accuracy, and precision. The method was applied to ten commercial cocoa husk tea products from Thailand. Theobromine was the predominant methylxanthine (10.483–16.027 mg g^−1^), whereas caffeine was lower (0.923–1.909 mg g^−1^), while flavanol contents varied among samples. These findings demonstrate that the developed method provides a rapid and reliable approach for the analysis and quality assessment of cocoa husk tea products and may support the further utilization of cocoa by-products in the functional beverage industry.

## 1. Introduction

The global cocoa industry generates substantial quantities of by-products during post-harvest processing, including cocoa bean husk, cocoa shell, and pulp. These materials account for approximately 85% of the total cocoa fruit mass and are traditionally regarded as low-value waste, contributing to environmental burdens and economic inefficiencies if not properly managed [1,2]. In recent years, however, increasing attention has been directed toward the valorization of cocoa by-products within the framework of sustainable food systems and circular economy principles. Among these by-products, cocoa husk has emerged as a particularly promising raw material due to its high content of bioactive compounds, including methylxanthines, polyphenols, dietary fiber, and phytosterols [1,2,3].

Cocoa husk contains a complex mixture of secondary metabolites that are known to exert a wide range of biological activities relevant to human health. Polyphenolic compounds, especially flavan-3-ols such as catechin and epicatechin, along with procyanidins, are widely recognized for their antioxidant, anti-inflammatory, cardioprotective, and metabolic regulatory properties [4,5,6]. In parallel, cocoa husk is a rich source of methylxanthines, with theobromine as the predominant compound and caffeine present in lower amounts. These alkaloids contribute not only to the characteristic bitterness and astringency of cocoa-derived products but also to physiological effects such as central nervous system stimulation, bronchodilation, and modulation of lipid metabolism [7,8,9].

Theobromine is typically the dominant methylxanthine in cocoa and cocoa by-products, occurring at concentrations several-fold higher than caffeine [3,7]. Compared with caffeine, theobromine exhibits milder stimulant effects and a longer half-life, making cocoa-derived beverages potentially attractive alternatives to conventional caffeinated drinks [8,9]. From a nutritional and functional perspective, the relatively high theobromine-to-caffeine ratio in cocoa husk may offer favorable sensory and physiological profiles, particularly in products intended for regular consumption.

Polyphenols in cocoa husk are predominantly represented by flavan-3-ols and their oligomeric forms, including procyanidin dimers such as procyanidin B1 and B2. These compounds are major contributors to the antioxidant capacity of cocoa matrices and have been implicated in protective effects against oxidative stress-related chronic diseases, including cardiovascular disease, type 2 diabetes, and certain cancers [4,5,10]. Nevertheless, the concentration and profile of these bioactive constituents are highly variable and depend on numerous factors, including cocoa genotype, geographical origin, agricultural practices, fermentation, drying, and roasting conditions [2,6].

In recent years, cocoa husk has attracted growing interest as a functional ingredient for incorporation into food products such as beverages, bakery items, chocolates, and dietary supplements. Several studies have demonstrated that cocoa husk extracts or powders can enhance antioxidant activity, dietary fiber content, and overall functional value of food matrices while simultaneously contributing to waste reduction and sustainability goals [1,3,11]. In particular, cocoa husk-based beverages, including cocoa husk tea, have gained popularity in niche markets due to their perceived health benefits and unique flavor profile. Despite this growing interest, comprehensive and standardized analytical data on the bioactive composition of commercial cocoa husk tea products remain limited.

Accurate determination of methylxanthines and polyphenolic compounds in cocoa husk-derived beverages is essential for quality control, nutritional labeling, and substantiation of health-related claims. High-performance liquid chromatography (HPLC), particularly when coupled with photodiode array (PDA) detection, is widely regarded as a robust and reliable analytical technique for the simultaneous determination of these compounds in complex food matrices [12,13]. Reversed-phase HPLC methods using C18 columns and acidified aqueous–organic mobile phases have been extensively applied for the separation and quantification of cocoa polyphenols and methylxanthines, owing to their high resolution, sensitivity, and reproducibility [13].

Method validation is a critical step to ensure the reliability and suitability of analytical procedures intended for routine application. International Conference on Harmonisation (ICH) guidelines (Q2(R2)) recommend that analytical methods be validated in terms of specificity, linearity, range, limits of detection (LOD), limits of quantification (LOQ), accuracy, and precision. Although numerous studies have reported HPLC-based analyses of cocoa beans and cocoa shell extracts, fewer investigations have focused specifically on cocoa husk tea prepared under standardized infusion conditions, such as those defined by ISO 3103:2019 for tea preparation. This gap in the literature limits the comparability of reported data and hampers the establishment of reference values for commercial products.

Moreover, most existing studies have concentrated on cocoa bean shells or laboratory-prepared extracts, often employing organic solvents or advanced extraction techniques such as microwave-assisted extraction, ultrasound-assisted extraction, or supercritical fluid extraction [1,3]. While these approaches are valuable for maximizing compound recovery, they may not accurately reflect the composition of cocoa husk tea as consumed by the general public. Hot water infusion remains the most relevant preparation method from a consumer perspective, yet systematic quantitative data on methylxanthines and individual polyphenols in commercially available cocoa husk tea products are still scarce. Thailand represents an emerging market for cocoa-based products, including cocoa husk tea, driven by increasing consumer interest in functional beverages and sustainable food innovations. However, variability in raw materials, processing conditions, and product formulations among local producers may lead to significant differences in bioactive compound content and, consequently, in potential health benefits. Comprehensive analytical evaluation of these products is therefore necessary to characterize their chemical profiles and to support product standardization and market development. In this context, the present study was designed to develop and validate a rapid, reliable, and sensitive HPLC–PDA method for the simultaneous determination of key methylxanthines (theobromine and caffeine) and selected polyphenolic compounds (catechin, epicatechin, procyanidin B1, and procyanidin B2) in cocoa husk tea. The method was validated in accordance with ICH Q2(R2) guidelines, assessing linearity, sensitivity, accuracy, precision, and specificity. Furthermore, the validated method was applied to quantify these bioactive compounds in ten commercial cocoa husk tea products obtained from different sources in Thailand, prepared using standardized infusion conditions based on ISO 3103:2019.

By focusing on commercially available cocoa husk tea products prepared under standardized hot-water infusion conditions according to ISO 3103:2019, this study provides comparative compositional data that more closely reflect actual consumer preparation practices. In addition, the validated HPLC–PDA method enabled the simultaneous determination of methylxanthines and selected polyphenolic compounds using a simple and practical analytical approach.

## 2. Results and Discussion

### 2.1. Specificity

The specificity of the proposed method was evaluated by comparing the chromatograms of the sample blank and spiked sample blank. Cocoa husk is known to be a complex matrix containing various classes of compounds, including polyphenols, methylxanthines, organic acids, and other degradation products generated during fermentation and roasting processes. The presence of these coexisting compounds may potentially interfere with chromatographic analysis. Therefore, evaluating method specificity is particularly important to ensure reliable identification and quantification of the target analytes in cocoa husk tea.

In the present study, no interfering peaks were observed at the retention times corresponding to the target analytes in the sample blank, while well-resolved and clearly identifiable peaks were detected in the spiked sample blank. These results demonstrate that the developed method possesses adequate selectivity and that the analytes can be distinguished from other matrix components.

Similar chromatographic behavior has been reported in previous studies employing reversed-phase HPLC for the analysis of cocoa polyphenols and related compounds [14]. For instance, reversed-phase chromatographic systems have been successfully applied for the separation of flavanols and phenolic compounds in cocoa-based products, providing well-resolved peaks suitable for quantitative analysis. The chromatographic characteristics observed in this study are consistent with these findings, supporting the suitability of reversed-phase HPLC for the determination of bioactive compounds in cocoa-derived matrices.

Although satisfactory chromatographic separation and PDA spectral confirmation were achieved, it should be noted that identification based solely on retention time and UV–Vis spectral characteristics may still have limitations in distinguishing structurally related isomers or unknown co-eluting compounds in complex matrices. Therefore, further confirmation using more advanced analytical techniques, such as LC–MS/MS, could provide enhanced specificity and structural confirmation of the target analytes.

**Figure 1:**
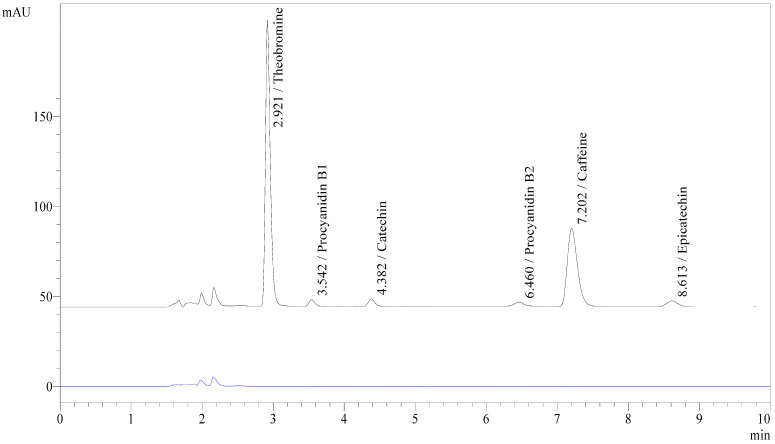
HPLC overlay chromatogram of the spiked sample blank and sample blank. The black line represents the spiked sample blank, while the blue line represents the sample blank.

Overall, the absence of interfering peaks from the sample matrix confirms that the developed method fulfills the selectivity requirements recommended by analytical method validation guidelines, such as ICH Q2(R2). This indicates that the method is suitable for the reliable quantitative determination of methylxanthines and flavanols in cocoa husk tea. An overlay chromatogram of the sample blank and spiked sample blank is presented in Figure 1.

### 2.2. Range and Linearity

The calibration ranges were selected based on the expected concentration levels of the target compounds in cocoa husk tea samples. These ranges were designed to adequately cover the anticipated levels of the analytes in the sample matrix, thereby ensuring reliable quantification without the need for extrapolation beyond the calibration limits.

Theobromine was calibrated over the concentration range of 15.625–500 ppm, whereas catechin, epicatechin, procyanidin B1, and procyanidin B2 were evaluated within the range of 3.125–100 ppm. In addition, caffeine was calibrated over the concentration range of 9.375–300 ppm. The selected calibration ranges were designed to reflect the relative abundance of bioactive compounds typically found in cocoa products. Cocoa is known to contain higher levels of methylxanthines, particularly theobromine, compared to caffeine, while flavanols such as catechin, epicatechin, and procyanidins are present as key polyphenolic compounds but generally at lower concentrations per individual compound. These compositional differences have been widely reported in cocoa and chocolate products [6,15,16].

The linearity of the method was evaluated using six concentration levels for each analyte corresponding to the selected calibration ranges. Each concentration level was analyzed in triplicate (*n* = 3) to enhance the reliability and reproducibility of the calibration model. Calibration curves were constructed by plotting peak area against the corresponding analyte concentration, and linear regression analysis was performed to obtain the regression equations and correlation coefficients (R^2^).

The results demonstrated excellent linearity for all target compounds, with correlation coefficients greater than 0.999. These values meet the commonly accepted criteria for quantitative analytical methods, particularly for HPLC-based determinations, where high coefficients of determination (R^2^) are indicative of good linearity, in accordance with current analytical method validation guidelines [17]. The use of six calibration levels analyzed in triplicate provided sufficient data points to support robust linear regression analysis and to ensure the reliability of the calibration model.

**Figure 2:**
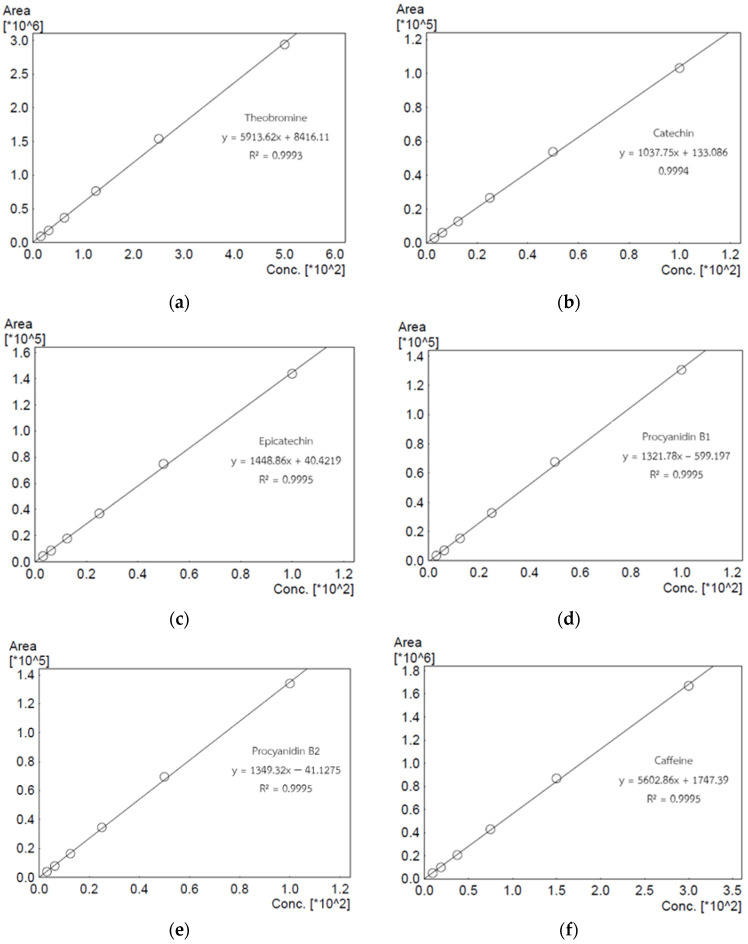
Calibration curves for the target analytes constructed from six concentration levels, each analyzed in triplicate (*n* = 3). The experimental design provides sufficient data points for reliable linear regression analysis and evaluation of method performance. (**a**) Theobromine. (**b**) Catechin. (**c**) Epicatechin. (**d**) Procyanidin B1. (**e**) Procyanidin B2. (**f**) Caffeine.

The regression equations, linearity data (R^2^) and repeatability parameters (%RSD) are summarized in Table 1, while the calibration curves for the target analytes are presented in Figure 2 and Supplementary Figures S1–S6. The low %RSD values obtained from triplicate measurements indicate good repeatability and consistency of the calibration responses across the tested concentration ranges confirming that the developed method is suitable for accurate quantification of methylxanthines and flavanol compounds in cocoa husk tea samples.

### 2.3. Limits of Detection (LODs) and Limits of Quantification (LOQs)

The limits of detection (LODs) and limits of quantification (LOQs) of the method were determined based on signal-to-noise (S/N) ratios and repeatability criteria. The LOD for each analyte was established at the concentration level providing a signal-to-noise ratio (S/N) equal to or greater than 3, based on six replicate measurements (*n* = 6). The LOQ was defined as the lowest concentration that could be quantified with acceptable repeatability, expressed as a percentage relative standard deviation (%RSD) of peak area ≤ 1%. In this study, the signal-to-noise ratios were obtained from chromatograms of diluted standard solutions analyzed under the optimized HPLC conditions. Representative chromatograms and signal-to-noise data used for LOD determination are provided in Supplementary Figures S7–S9 and Tables S1–S3, while chromatograms and peak area data used for LOQ determination are presented in Supplementary Figures S10–S12 and Tables S4–S6.

The LOD and LOQ values for theobromine were 0.386 ppm and 0.732 ppm, respectively, corresponding to signal-to-noise ratios greater than 3 and %RSD values ≤ 1%. For catechin, epicatechin, procyanidin B1, and procyanidin B2, the LOD and LOQ values were 3.125 ppm and 6.250 ppm, respectively, satisfying the criteria of S/N ≥ 3 and %RSD ≤ 1%. Similarly, caffeine exhibited LOD and LOQ values of 0.800 ppm and 1.510 ppm, respectively, meeting the same acceptance criteria.

The relatively low detection limits obtained in this study indicate that the developed HPLC-PDA method provides sufficient sensitivity for the determination of methylxanthines and flavanol compounds in cocoa husk tea samples. Similar analytical approaches using HPLC with diode-array detection have been widely applied for the determination of methylxanthines in food and beverage matrices.

Previous studies that have reported simultaneous HPLC methods for the determination of caffeine, theobromine, and theophylline in foods and beverages have reported detection limits in the sub-mg L^−1^ to low mg L^−1^ range, which are comparable to the sensitivity achieved in the present study. These findings indicate that the detection capability of the developed method is consistent with previously reported HPLC methods for methylxanthine analysis in food systems [18].

Therefore, the obtained LOD and LOQ values demonstrate that the developed method possesses adequate sensitivity and precision for the quantitative determination of the target compounds. The analytical performance of the method supports its applicability for routine compositional analysis and quality control of cocoa husk tea products. The calculated LOD and LOQ values are summarized in Table 2.

### 2.4. Accuracy and Precision

The accuracy of the analytical method was determined by spiking pre-analyzed samples with known amounts of the standards at low, medium, and high concentration levels within the calibration range. The spiked samples were analyzed using the proposed method in six replicates (*n* = 6).

The percentage recoveries of the target analytes were evaluated according to the criteria specified in Appendix F: Guidelines for Standard Method Performance Requirements [19], and the results are summarized in Table 3. The acceptance criteria for recovery and detailed recovery data obtained from spiked samples at low (QL), medium (QM), and high (QH) concentration levels are provided in Supplementary Table S7 and Tables S8–S25, respectively. The recovery values obtained for all analytes ranged from 82.27% to 107.21%, indicating acceptable analytical accuracy. According to these guidelines, acceptable recovery ranges vary depending on the analyte concentration, with typical values of 80–110% reported for low concentration levels. Therefore, the recovery results obtained in this study demonstrate that the proposed HPLC method provides reliable quantitative performance for the determination of methylxanthines and polyphenolic compounds in cocoa husk tea.

Slightly lower recovery values were observed at the lowest spiking levels for some analytes, particularly theobromine and catechin. This phenomenon may be attributed to several factors, including matrix effects from the cocoa husk infusion, minor losses during extraction, or reduced detector response at very low concentrations. Cocoa-derived matrices are known to contain a wide range of co-extracted compounds, including flavanols, methylxanthines, and other phenolic constituents, which can influence analyte recovery and signal response during chromatographic analysis.

In addition, the extraction efficiency of phenolic compounds can be influenced by their chemical structure and interactions with the sample matrix, potentially resulting in slight analyte losses during sample preparation. Theobromine and catechin, which differ in polarity and solubility characteristics, may therefore be affected differently under the applied extraction and analytical conditions. Similar observations have been reported in the analysis of cocoa and other plant-derived products [6].

The precision of the proposed method was evaluated in terms of intra-day and inter-day variability using a real cocoa husk tea sample, and the results are summarized in Table 4 and Table 5. The acceptance criteria for precision are presented in Supplementary Table S26, while detailed intra-day precision data and inter-day precision data are provided in Supplementary Tables S27–S31 and Tables S32–S36, respectively. Intra-day precision was assessed by analyzing six replicates within a single day, while inter-day precision was evaluated over three consecutive days with six replicates per day under identical experimental conditions. Precision was expressed as the percentage relative standard deviation (%RSD) of retention time and concentration, and evaluated according to the acceptance criteria specified in Appendix F: Guidelines for Standard Method Performance Requirements [19].

For intra-day precision, the %RSD values for retention time ranged from 0.043% to 0.099%, indicating excellent repeatability and system stability. The %RSD values for concentration ranged from 0.852% to 5.147%. All %RSD values were within acceptable limits, demonstrating good precision of the method. Slightly higher variability was observed for epicatechin, which may be attributed to its relatively low concentration and potential matrix effects, whereas theobromine showed more consistent results due to its higher abundance.

For inter-day precision, the %RSD values for retention time ranged from 0.131% to 0.551%, confirming good reproducibility over multiple days. The %RSD values for concentration ranged from 0.820% to 4.769%, and all values were within acceptable limits. Catechin and epicatechin exhibited relatively higher variability compared to other analytes, likely due to their lower concentrations and susceptibility to matrix interferences. In contrast, theobromine and caffeine showed lower variability, indicating stable quantification across different days. The lower variability observed for theobromine and caffeine is consistent with previous studies, which report that methylxanthines present at higher concentrations generally provide more stable detector responses and improved precision [20].

Overall, the results demonstrate that the developed HPLC method is precise, reliable, and reproducible for the determination of methylxanthines and polyphenols in cocoa husk tea, with all %RSD values complying with the recommended acceptance criteria.

### 2.5. Application of Methylxanthine and Polyphenols in Cocoa Husk Tea from Different Commercial Sources in Thailand

**Figure 3:**
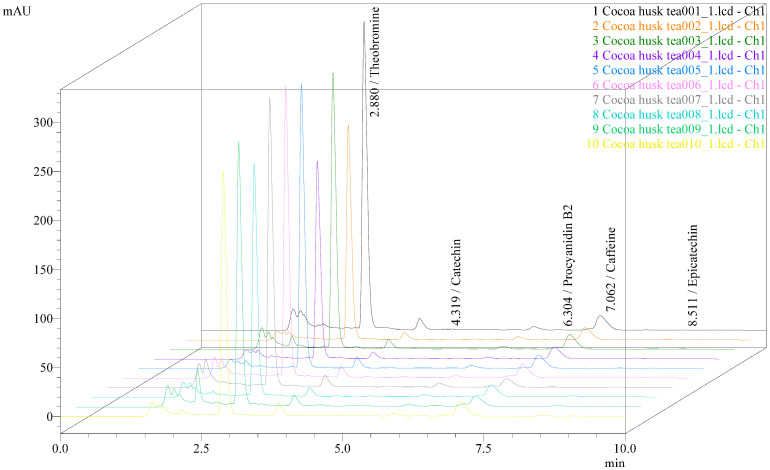
The overlay chromatograms of commercial cocoa husk tea samples from different brands in Thailand (*n* = 10).

The contents of methylxanthines and selected polyphenolic compounds in 10 commercial cocoa husk tea samples obtained from different sources in Thailand are summarized in Table 6 and Figure 3. The concentrations of individual compounds were determined from the infusion samples and subsequently converted and expressed as mg/g based on the dry weight of the original cocoa husk tea products. The results reveal noticeable variability in the levels of bioactive compounds among the analyzed samples. Such differences may reflect variations in raw materials, cocoa cultivar, geographical origin, and post-harvest processing conditions, including fermentation, drying, and roasting. Similar variability in the chemical composition of cocoa by-products and cocoa-derived beverages has been widely reported in previous studies [1,2,3].

Theobromine was consistently identified as the predominant methylxanthine in all samples, with concentrations ranging from 10.483 to 16.027 mg/g, whereas caffeine was present at markedly lower levels (0.923–1.909 mg/g). This distribution is in good agreement with earlier reports on cocoa shells and husks, which consistently describe theobromine as the major alkaloid due to its higher biosynthetic accumulation in cocoa tissues compared to caffeine [3,6,7]. The relatively high theobromine-to-caffeine ratio observed in the present study supports the notion that cocoa husk-based beverages may exert milder stimulant effects than conventional caffeinated drinks, such as coffee or traditional tea [6].

Variations in methylxanthine content among the samples may be attributed to several factors, including cocoa cultivar, geographical origin, and post-harvest processing. Fermentation and roasting, in particular, have been reported to significantly influence methylxanthine levels by promoting diffusion, thermal degradation, or migration of alkaloids between cocoa bean fractions and surrounding tissues [6,21]. Moreover, the water solubility of methylxanthines facilitates their extraction during hot-water infusion, although extraction efficiency may vary depending on particle size, husk thickness, and degree of cell wall disruption caused by prior processing steps [13].

In contrast to methylxanthines, polyphenolic compounds exhibited greater variability across the commercial samples. Catechin, epicatechin, and procyanidin B2 were detected at concentrations ranging from trace levels to 0.740 mg/g, and were not consistently present in all samples. This pronounced variation is consistent with previous findings indicating that cocoa polyphenols are highly sensitive to fermentation, drying, and roasting conditions [2,10]. During fermentation, enzymatic oxidation mediated by polyphenol oxidase can lead to substantial losses of monomeric flavan-3-ols, while roasting may further promote thermal degradation and polymerization reactions [11].

Procyanidins, particularly oligomeric forms such as procyanidin B1 and B2, are known to be less stable than monomeric catechins under processing conditions and during aqueous extraction [22]. The higher variability observed for procyanidin B2 compared to catechin and epicatechin may be related to differences in the degree of polymerization and susceptibility to depolymerization or oxidative cleavage [13]. Additionally, hot-water infusion, while representative of consumer preparation, is generally less efficient for extracting higher-molecular-weight polyphenols compared to organic solvent-based extraction methods commonly used in laboratory studies [3]. Brewing conditions, including water temperature, infusion time, and solid-to-water ratio, can significantly influence the extraction efficiency of bioactive compounds in tea beverages. Therefore, the ISO 3103:2019 hot-water infusion method was employed in this study to provide a standardized and reproducible brewing procedure, although it may not fully reflect variations in individual consumer preparation practices [23].

Procyanidin B1 was not detected in any of the analyzed samples. This observation is consistent with reports indicating that procyanidin B1 typically occurs at lower concentrations in cocoa husk compared to cocoa beans and may fall below detection limits when infusion-based extraction is applied [2,9]. Furthermore, differences in stereochemistry and linkage type between procyanidin dimers have been shown to influence their extractability and chromatographic detectability [13].

Noticeable variations in the composition of bioactive compounds were observed among the commercial cocoa husk tea brands analyzed in this study. Theobromine was consistently the predominant methylxanthine in all samples. Brand no. 1 exhibited the highest theobromine content, whereas Brand no. 4 showed the lowest level. In contrast, caffeine concentrations were substantially lower across all samples, confirming the characteristic methylxanthine profile commonly reported for cocoa-derived products.

The calculated theobromine/caffeine ratios varied among brands, ranging approximately from 7.7 to 15.1. Brand no. 9 showed the highest ratio, while Brand no. 1 exhibited the lowest ratio despite having the highest absolute theobromine concentration. These differences suggest that the relative accumulation and extraction of methylxanthines may vary depending on cocoa origin, processing conditions, and manufacturing practices. From a consumer perspective, the relatively high theobromine-to-caffeine ratios observed in all samples may contribute to milder stimulant effects compared with conventional coffee or tea beverages, potentially making cocoa husk tea a suitable alternative for consumers seeking reduced caffeine intake while still obtaining bioactive methylxanthines [5,7].

Overall, the results demonstrate that while cocoa husk tea represents a consistent source of methylxanthines, particularly theobromine, its polyphenolic composition is highly dependent on processing and raw material variability. These findings highlight the importance of standardized processing and analytical methodologies for quality control and support the previous literature emphasizing the heterogeneous nature of bioactive compounds in cocoa by-products [1,3,10]. The application of a validated HPLC–PDA method using standardized infusion conditions, as presented in this study, provides a reliable framework for the comparative assessment of commercial cocoa husk tea products.

## 3. Materials and Methods

### 3.1. Chemicals and Reagents

The standards of theobromine, catechin, epicatechin, procyanidin B1 and procyanidin B2 were purchased from ChemFaces (Wuhan, China) and caffeine was purchased from Sigma-Aldrich (St. Louis, MO, USA). Acetonitrile (HPLC grade) was obtained from Sigma-Aldrich (St. Louis, MO, USA). Methanol (HPLC grade) was purchased from JT Baker (Phillipsburg, NJ, USA). Glacial acetic acid (AR grade) was obtained from QReC (New Zealand), and DMSO (analytical reagent grade) was purchased from Fisher Chemical (Waltham, MA, USA). Ultrapure water (resistivity at 25 °C, 18.2 MΩ·cm) was used throughout the experiments.

### 3.2. Instrument Used

A high-performance liquid chromatography system operating under a gradient elution mode was used in this study. The system consisted of a Shimadzu LC-40D XR pump, an SIL-40C XR autosampler, and an SPD-M40 photodiode array (PDA) detector (Shimadzu Corporation, Kyoto, Japan). LabSolutions software version 5.117 (Shimadzu Corporation, Kyoto, Japan) was used for data acquisition and processing. Other laboratory instruments included a centrifuge (Hettich Zentrifugen, Tuttlingen, Germany), a vortex mixer (DLAB Scientific, Beijing, China), a vacuum pump (Millipore, Burlington, MA, USA), an analytical balance (OHAUS Corporation, Parsippany, NJ, USA), and micropipettes (Discovery Comfort). A nylon membrane filter (0.45 µm) was used during sample preparation.

### 3.3. HPLC Instrumentation and Conditions

High-performance liquid chromatography was used for the determination of methylxanthines and flavanols in cocoa husk tea samples. Analyses were performed using a Shimadzu Nexera LC-40 system with PDA detector. Separation was realized on a reverse-phase column (Zorbax 300SB-C18, 4.6 × 150 mm, 3.5 µm particle size, Agilent Technologies, Santa Clara, CA, USA). The column temperature was maintained at 40 °C. The mobile phases were 2% acetic acid in water (A) and acetonitrile (B). A total run time of 15 min: initially, 95% A for 7.50 min, to 20% A in 1 min (at 8.50 min), which was held until 10.50 min. The gradient was then returned to 95% at 11.00 min and maintained until 15.00 min for column re-equilibration. The flow rate was 1 mL min^−1^. The detection wavelength in UV was 280 nm. The injection volume was 2 µL.

### 3.4. Preparation of Standard Solutions

Stock standard solutions of theobromine, catechin, epicatechin, procyanidin B1, procyanidin B2, and caffeine were prepared by accurately weighing each compound and dissolving them in an appropriate solvent (DMSO followed by dilution with methanol) to obtain individual stock concentrations of 1 mg/mL. These stock solutions were subsequently diluted with methanol to prepare a series of working standard solutions for calibration.

Working solutions for theobromine were prepared at concentrations of 500, 250, 125, 62.5, 31.25 and 15.625 ppm. Working solutions for catechin, epicatechin, procyanidin B1, and procyanidin B2 were prepared at 100, 50, 25, 12.5, 6.25 and 3.125 ppm. Working solutions for caffeine were prepared at 300, 150, 75, 37.5, 18.75 and 9.375 ppm. All solutions were vortexed until completely dissolved, filtered through a 0.45 µm nylon membrane filter, and stored at 4 °C until use.

### 3.5. Preparation of Samples

A total of 10 commercial cocoa husk tea samples, representing various local brands, were obtained from chocolate-oriented online retail shops and selected cafés in Thailand. Sample preparation for analysis was performed in accordance with ISO 3103:2019, International Organization for Standardization, 2019 [24]. The cocoa husk tea samples were ground to obtain a homogeneous consistency, after which approximately 2 g. of the sample was weighed into a heat-resistant container. Hot water (100 mL) at a temperature of approximately 80–95 °C was added, and the mixture was infused for 6 min. The filtration of the sample was done using a nylon membrane filter of 0.45 µm size before being injected into the system of HPLC system for analysis.

### 3.6. Method Validation

As recommended by International Council for Harmonisation (ICH) guidelines [17], the validation characteristics considered in this study included specificity, range, linearity, limits of detection (LOD) and quantification (LOQ), accuracy, and precision.

#### 3.6.1. Specificity of the Method

Specificity was evaluated by analyzing both the sample blank and the spiked sample blank. Chromatograms of these samples were compared to assess potential interferences from matrix components at the retention times of the target analytes.

#### 3.6.2. Evaluation of Range and Linearity

The linearity of the method was evaluated by preparing standard solutions at six different concentrations within the expected range. The calibration curve was constructed by plotting the peak area versus concentration, and the correlation coefficient (R^2^) was used to assess linearity.

#### 3.6.3. Determination of Limits of Detection and Quantification

The analytical sensitivity of the method was evaluated in terms of the limit of detection (LOD) and limit of quantitation (LOQ). LOD was determined based on a signal-to-noise ratio (S/N) of 3. LOQ was defined as the lowest concentration that could be reliably quantified with acceptable repeatability, expressed as %RSD ≤ 1%.

#### 3.6.4. Accuracy and Precision Assessment

Accuracy was determined by analyzing samples spiked with known concentrations of the standards at low, medium, and high levels. Recovery (%) was calculated by comparing the measured concentrations with the corresponding added concentrations. Precision was evaluated in terms of repeatability (intra-day) and intermediate precision (inter-day) by analyzing six replicate measurements of the same sample under the same conditions. The results were expressed as relative standard deviation (%RSD).

## 4. Conclusions

A rapid and reliable HPLC–PDA method was successfully developed and validated for the simultaneous determination of methylxanthines and selected polyphenolic compounds in cocoa husk tea. The method demonstrated good specificity, excellent linearity (R^2^ > 0.999), satisfactory sensitivity, accuracy, and precision, confirming its suitability for routine analysis and quality control. Application of the method to commercial cocoa husk tea samples from different sources in Thailand showed that theobromine was the predominant methylxanthine, while caffeine and polyphenolic compounds, including catechin, epicatechin, and procyanidin B2, were present at varying levels depending on the source. Overall, the results support the potential of cocoa husk tea as a functional beverage and highlight the valorization of cocoa husk as a value-added by-product within the cocoa processing chain.

Future studies may employ advanced analytical techniques such as LC–MS/MS for more comprehensive characterization and metabolomic profiling of bioactive compounds in cocoa husk tea. In addition, further studies involving broader compound profiling, monitoring the effects of processing conditions, and evaluating a wider range of cocoa husk samples may provide deeper insight into compositional variations and support the development of standardized cocoa husk tea products.

## Figures and Tables

**Table 1:** Calibration range, linearity, and repeatability parameter.

Compound	Range (ppm)	%RSD Range	R^2^
Theobromine	15.625–500	0.041–0.563	0.9993
Catechin	3.125–100	0.063–0.539	0.9994
Epicatechin	3.125–100	0.086–0.689	0.9995
Procyanidin B1	3.125–100	0.112–0.754	0.9995
Procyanidin B2	3.125–100	0.094–3.142	0.9995
Caffeine	9.375–300	0.039–0.393	0.9995

**Table 2:** LOD and LOQ values of individual standard compounds.

Compound	LOD (ppm)	LOQ (ppm)
Theobromine	0.386	0.732
Catechin	3.125	6.250
Epicatechin	3.125	6.250
Procyanidin B1	3.125	6.250
Procyanidin B2	3.125	6.250
Caffeine	0.800	1.510

**Table 3:** Recovery studies (*n* = 6).

Compound	Concentration of Spiked (ppm)	% Recovery
	15.625	84.52
Theobromine	125	101.15
	500	99.91
	3.125	82.27
Catechin	25	101.58
	100	99.66
	3.125	90.12
Epicatechin	25	100.63
	100	99.76
	3.125	106.32
Procyanidin B1	25	95.79
	100	100.12
	3.125	107.20
Procyanidin B2	25	104.63
	100	98.90
	9.375	89.09
Caffeine	75	100.77
	300	99.98

**Table 4:** Intra-day precision of the retention time and concentration of methylxanthine and polyphenols in cocoa husk tea (*n* = 6).

Compound	Retention Time		Concentration	
	min	%RSD	mg g^−1^	%RSD
Theobromine	2.897	0.091	17.363	0.852
Catechin	4.340	0.066	0.140	1.978
Epicatechin	8.555	0.043	0.174	5.147
Procyanidin B2	6.341	0.099	0.317	3.137
Caffeine	7.120	0.049	1.761	1.348

**Table 5:** Inter-day precision (3 days) of the retention time and concentration of methylxanthine and polyphenols in cocoa husk tea (*n* = 6).

Compound	Retention Time		Concentration	
	min	%RSD	mg g^−1^	%RSD
Theobromine	2.906	0.322	17.360	0.820
Catechin	4.350	0.267	0.133	4.769
Epicatechin	8.580	0.408	0.173	4.465
Procyanidin B2	6.340	0.131	0.314	3.037
Caffeine	7.159	0.551	1.776	1.313

**Table 6:** Concentrations of bioactive compounds in commercial cocoa husk tea samples from different brands in Thailand (mg g^−1^).

Brand No.	Theobromine	Catechin	Procyanidin B2	Caffeine	Epicatechin
Brand no. 1	16.027 ± 0.3060 ^a^	0.124 ± 0.0006 ^g^	0.212 ± 0.0070 ^e^	1.909 ± 0.0325 ^a^	0.146 ± 0.0055 ^h^
Brand no. 2	11.526 ± 0.0240 ^f^	0.000 ± 0.0000 ^h^	0.213 ± 0.0090 ^e^	1.505 ± 0.0085 ^c^	0.237 ± 0.0110 ^e^
Brand no. 3	15.195 ± 0.5345 ^b^	0.331 ± 0.0030 ^e^	0.215 ± 0.0060 ^e^	1.683 ± 0.0640 ^b^	0.202 ± 0.0050 ^f^
Brand no. 4	10.483 ± 0.0635 ^g^	0.000 ± 0.0000 ^h^	0.356 ± 0.0070 ^d^	1.420 ± 0.0125 ^d^	0.471 ± 0.0095 ^b^
Brand no. 5	15.172 ± 0.0595 ^b^	0.000 ± 0.0000 ^h^	0.142 ± 0.0045 ^f^	1.617 ± 0.0035 ^b^	0.179 ± 0.0030 ^g^
Brand no. 6	15.660 ± 0.3435 ^ab^	0.396 ± 0.0020 ^c^	0.101 ± 0.0020 ^g^	1.422 ± 0.0370 ^d^	0.071 ± 0.0015 ^i^
Brand no. 7	15.257 ± 0.0205 ^b^	0.231 ± 0.0015 ^f^	0.000 ± 0.0000 ^h^	0.923 ± 0.0060 ^f^	0.000 ± 0.0000 ^j^
Brand no. 8	12.206 ± 0.0200 ^e^	0.449 ± 0.0035 ^b^	0.584 ± 0.0025 ^b^	1.359 ± 0.0060 ^d^	0.645 ± 0.0040 ^a^
Brand no. 9	13.924 ± 0.0125 ^c^	0.627 ± 0.0050 ^a^	0.740 ± 0.0010 ^a^	1.006 ± 0.0010 ^e^	0.433 ± 0.0040 ^c^
Brand no. 10	13.016 ± 0.0380 ^d^	0.378 ± 0.0035 ^d^	0.380 ± 0.0035 ^c^	1.649 ± 0.0045 ^b^	0.364 ± 0.0045 ^d^

Values are expressed as mean ± SD. Different lowercase superscript letters within the same column indicate significant differences at *p*-value < 0.05 according to Tukey’s multiple comparison test.

## Data Availability

The data supporting the findings of this study are available within the article and its Supplementary Materials.

## Supplementary Materials

The following supporting information can be downloaded at: https://www.mdpi.com/article/10.3390/molecules31101697/s1, Figures S1–S6: Calibration curves of the target analytes; Figures S7–S9 and Tables S1–S3: Chromatograms and signal-to-noise data for determine LOD; Figures S10–S12 and Tables S4–S6: Chromatograms and Peak area for determine LOQ; Table S7 and Tables S8–S25: Acceptance criteria for %recovery and %recovery from spiked at QL, QM and QH; Table S26 and Tables S27–S31: Acceptance criteria for precision and Intra-day precision in cocoa husk tea; and Tables S32–S36: Inter-day precision in cocoa husk tea.
