# Supplementary material for: Development and Validation of a Rapid High-Performance Liquid Chromatography Method for Simultaneous Determination of Methylxanthines and Flavanols in Cocoa Husk Tea
Source: Molecules. 2026 May 17;31(10):1697. doi: 10.3390/molecules31101697 (PMC13209721; doi:10.3390/molecules31101697)
Supplement: Supplementary file 1 [file molecules-31-01697-s001.zip › Figures S10–S12 and Tables S4-S6. Chromatograms and Peak area for determine LOQ.pdf]

## Supplementary Materials

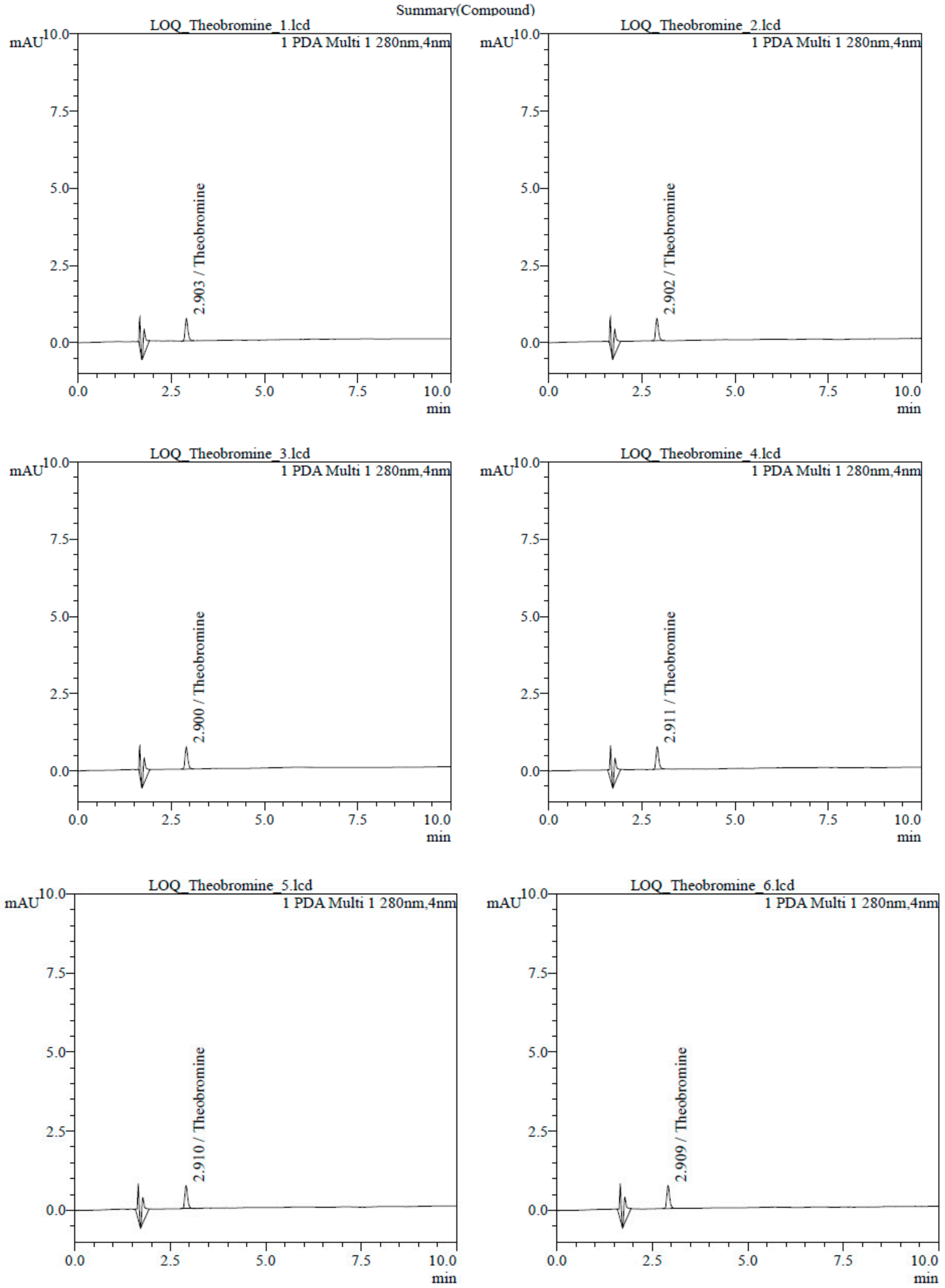


**Figure S10.** Chromatograms of Theobromine at the limit of quantification (n=6)

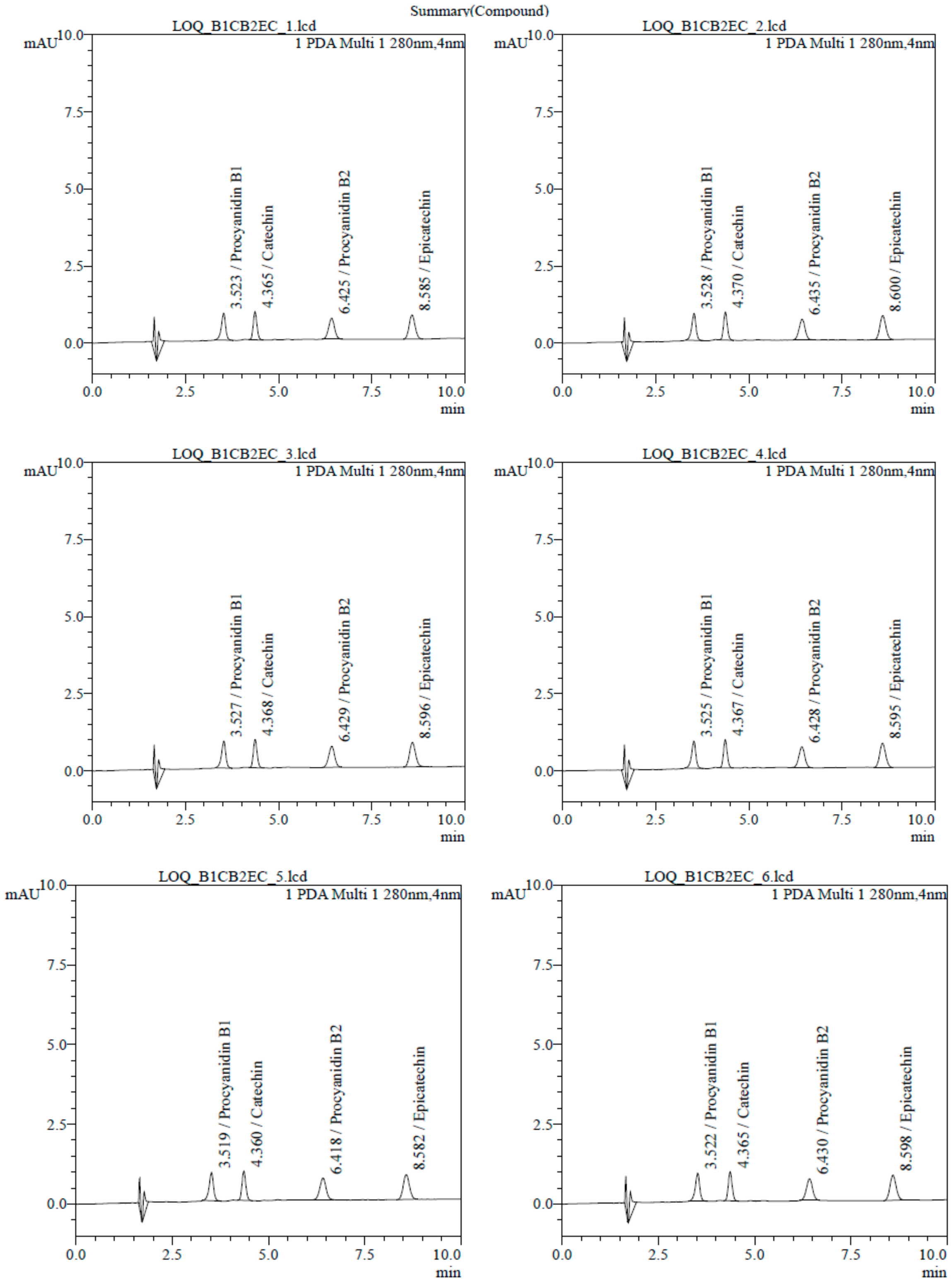


**Figure S11.** Chromatograms of Catechin, Epicatechin, Procyanidin B1, and Procyanidin B2 at the limit of quantification (n=6)

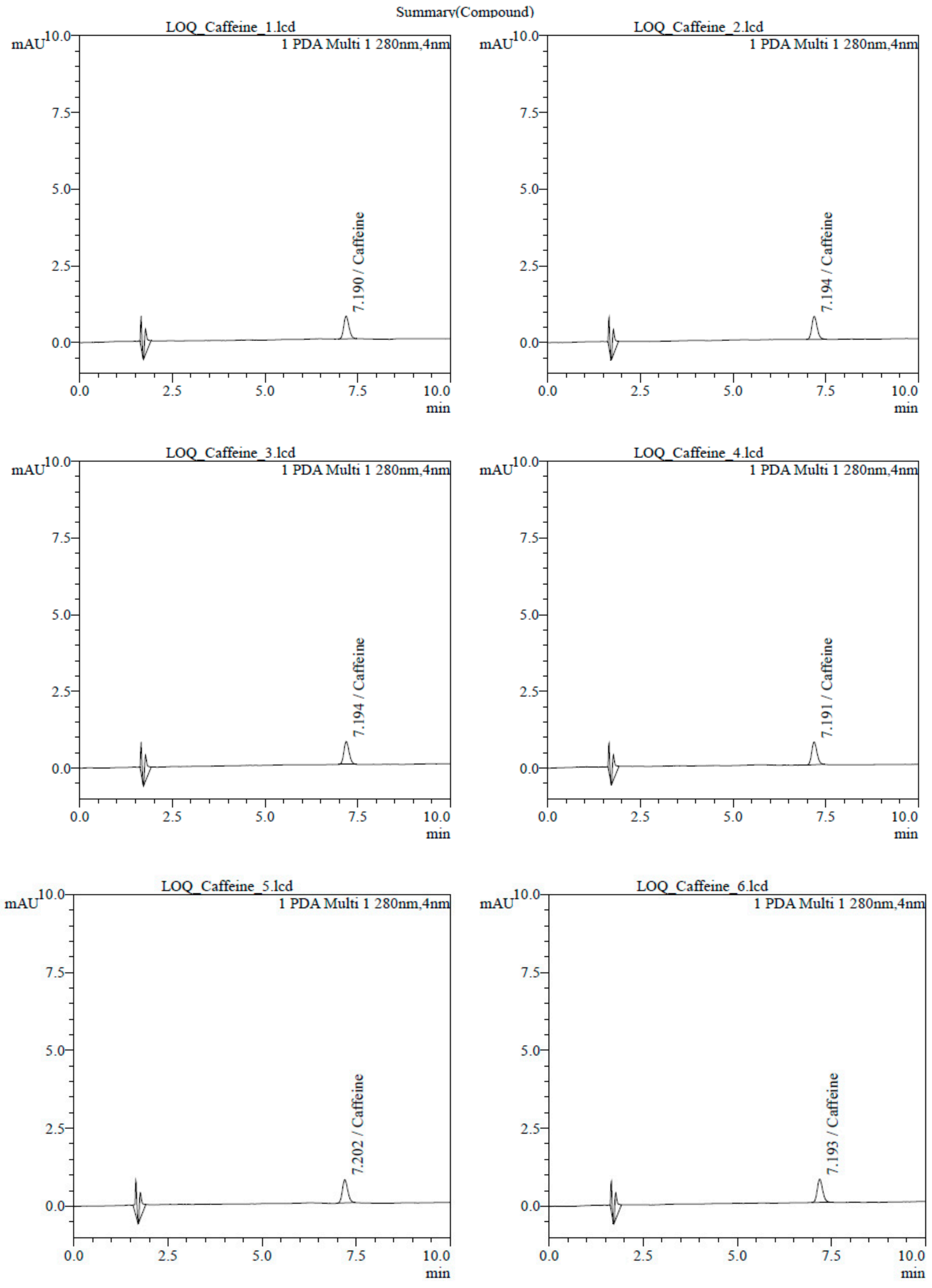


**Figure S12.** Chromatograms of Caffeine at the limit of quantification (n=6)

**Table S4.** Peak area of Theobromine at 0.732 ppm (n=6)

| Sample name | Area |
|---|---|
| LOQ_Theobromine_1 | 3,657 |
| LOQ_Theobromine_2 | 3,698 |
| LOQ_Theobromine_3 | 3,643 |
| LOQ_Theobromine_4 | 3,637 |
| LOQ_Theobromine_5 | 3,686 |
| LOQ_Theobromine_6 | 3,689 |
| **Average** | 3,668 |
| **SD** | 25.97 |
| **%RSD** | **0.71** |
| **%RSD ≤ 1** | **Pass** |

**Table S5.** Peak area of Catechin, Epicatechin, Procyanidin B1, and Procyanidin B2 at 6.250 ppm (n=6)

| Sample name | Area | Average | SD | %RSD | %RSD ≤ 1 |
|---|---|---|---|---|---|
| LOQ_Catechin_1 | 6,318 | 6,370 | 45.50 | **0.71** | **Pass** |
| LOQ_Catechin_2 | 6,346 | | | | |
| LOQ_Catechin_3 | 6,439 | | | | |
| LOQ_Catechin_4 | 6,360 | | | | |
| LOQ_Catechin_5 | 6,411 | | | | |
| LOQ_Catechin_6 | 6,347 | | | | |
| LOQ_Epicatechin_1 | 8,746 | 8,770 | 64.09 | **0.73** | **Pass** |
| LOQ_ Epicatechin _2 | 8,738 | | | | |
| LOQ_ Epicatechin _3 | 8,871 | | | | |
| LOQ_ Epicatechin _4 | 8,702 | | | | |
| LOQ_ Epicatechin _5 | 8,737 | | | | |
| LOQ_ Epicatechin _6 | 8,825 | | | | |
| LOQ_ Procyanidin B1 _1 | 7,015 | 7,041 | 71.91 | **1.02** | **Pass** |
| LOQ_ Procyanidin B1 _2 | 7,022 | | | | |
| LOQ_ Procyanidin B1 _3 | 6,923 | | | | |
| LOQ_ Procyanidin B1 _4 | 7,100 | | | | |
| LOQ_ Procyanidin B1 _5 | 7,060 | | | | |
| LOQ_ Procyanidin B1 _6 | 7,125 | | | | |
| LOQ_ Procyanidin B2 _1 | 7,574 | 7,558 | 74.48 | **0.99** | **Pass** |
| LOQ_ Procyanidin B2 _2 | 7,437 | | | | |
| LOQ_ Procyanidin B2 _3 | 7,648 | | | | |
| LOQ_ Procyanidin B2 _4 | 7,531 | | | | |
| LOQ_ Procyanidin B2 _5 | 7,619 | | | | |
| LOQ_ Procyanidin B2 _6 | 7,541 | | | | |

**Table S6.** Peak area of Caffeine at 1.510 ppm (n=6)

| **Sample name** | **Area** |
|---|---|
| LOQ_ Caffeine _1 | 7,352 |
| LOQ_ Caffeine _2 | 7,498 |
| LOQ_ Caffeine _3 | 7,365 |
| LOQ_ Caffeine_4 | 7,385 |
| LOQ_ Caffeine _5 | 7,373 |
| LOQ_ Caffeine _6 | 7,410 |
| **Average** | 7,397 |
| **SD** | 53.18 |
| **%RSD** | **0.72** |
| **%RSD ≤ 1** | **Pass** |
