# Supplementary material for: Development and Validation of a Rapid High-Performance Liquid Chromatography Method for Simultaneous Determination of Methylxanthines and Flavanols in Cocoa Husk Tea
Source: Molecules. 2026 May 17;31(10):1697. doi: 10.3390/molecules31101697 (PMC13209721; doi:10.3390/molecules31101697)
Supplement: Supplementary file 1 [file molecules-31-01697-s001.zip › Figures S1–S6. Calibration curves of the target analytes.pdf]

Supplementary Materials

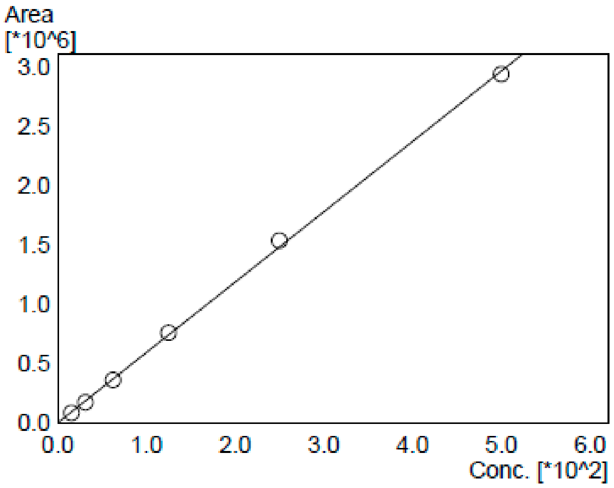

#	Conc.(Ratio)	MeanArea	Area
1	15.625	88818	88653
			88421
			89379
2	31.25	177445	177245
			177218
			177872
3	62.5	365440	365400
			365927
			364993
4	125	762394	762208
			762752
			762223
5	250	1539486	1539651
			1538405
			1540400
6	500	2938131	2937316
			2937298
			2939779

Figure S1. Calibration curve of Theobromine constructed using six concentration levels (ppm), each analyzed in triplicate (n = 3). The regression equation was y = 5913.62x + 8416.11 with a coefficient of determination (R^2) of 0.9993

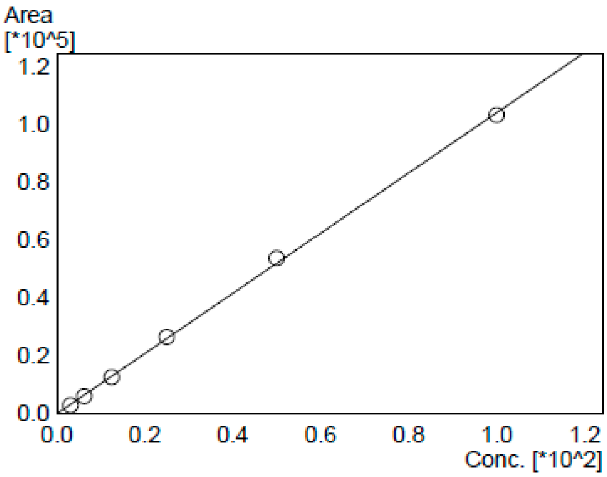

#	Conc.(Ratio)	MeanArea	Area
1	3.125	2999	3007
			2989
			3001
2	6.25	6128	6122
			6098
			6163
3	12.5	12660	12648
			12694
			12639
4	25	26571	26602
			26553
			26558
5	50	53709	53725
			53593
			53808
6	100	103038	102971
			103045
			103100

Figure S2. Calibration curve of Catechin constructed using six concentration levels (ppm), each analyzed in triplicate (n = 3). The regression equation was y = 1037.75x + 133.086 with a coefficient of determination (R^2) of 0.9994

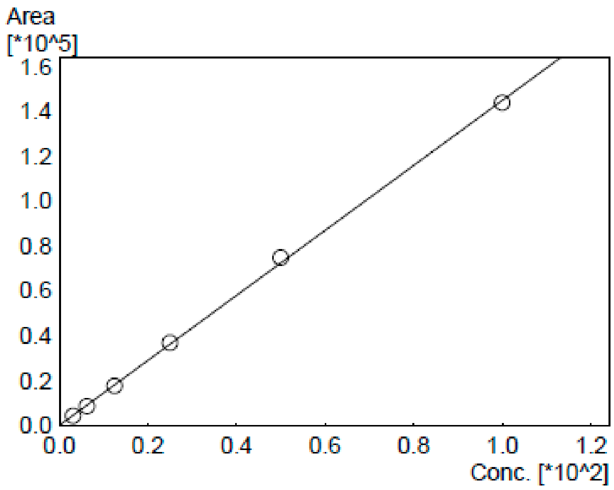

#	Conc.(Ratio)	MeanArea	Area
1	3.125	4178	4152
			4177
			4205
2	6.25	8451	8503
			8388
			8460
3	12.5	17573	17592
			17544
			17583
4	25	36774	36748
			36850
			36723
5	50	74694	74833
			74589
			74660
6	100	143818	143724
			143773
			143958

Figure S3. Calibration curve of Epicatechin constructed using six concentration levels (ppm), each analyzed in triplicate (n = 3). The regression equation was y = 1448.86x + 40.4219 with a coefficient of determination (R^2) of 0.9995

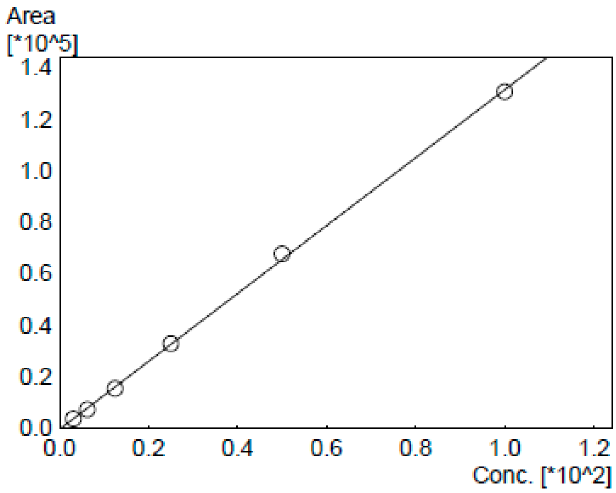

#	Conc.(Ratio)	MeanArea	Area
1	3.125	3425	3430
			3440
			3404
2	6.25	7141	7196
			7088
			7138
3	12.5	15255	15200
			15191
			15375
4	25	32658	32780
			32656
			32538
5	50	67522	67553
			67377
			67636
6	100	130628	130664
			130467
			130753

Figure S4. Calibration curve of Procyanidin B1 constructed using six concentration levels (ppm), each analyzed in triplicate (n = 3). The regression equation was y = 1321.78x - 599.197 with a coefficient of determination (R^2) of 0.9995

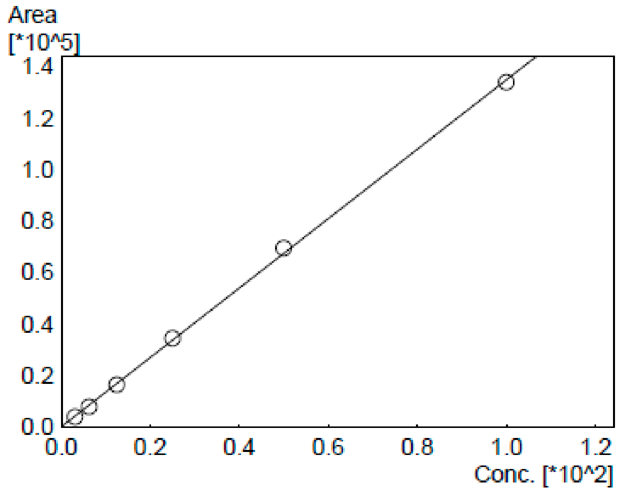

#	Conc.(Ratio)	MeanArea	Area
1	3.125	3799	3703
			3760
			3932
2	6.25	7756	7687
			7825
			7756
3	12.5	16246	16270
			16300
			16167
4	25	34306	34338
			34368
			34213
5	50	69441	69371
			69453
			69500
6	100	133853	133821
			133708
			134030

Figure S5. Calibration curve of Procyanidin B2 constructed using six concentration levels (ppm), each analyzed in triplicate (n = 3). The regression equation was y = 1349.32x - 41.1275 with a coefficient of determination (R^2) of 0.9995

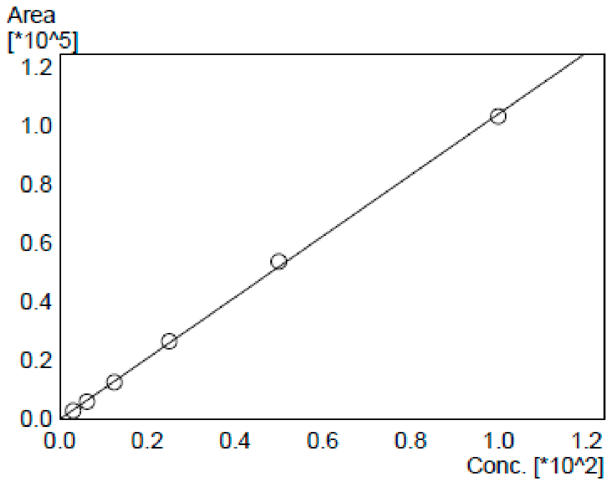

#	Conc.(Ratio)	MeanArea	Area
1	3.125	2999	3007
			2989
			3001
2	6.25	6128	6122
			6098
			6163
3	12.5	12660	12648
			12694
			12639
4	25	26571	26602
			26553
			26558
5	50	53709	53725
			53593
			53808
6	100	103038	102971
			103045
			103100

Figure S6. Calibration curve of Caffeine constructed using six concentration levels (ppm), each analyzed in triplicate (n = 3). The regression equation was y = 5602.86x + 1747.39 with a coefficient of determination (R^2) of 0.9995
