# Supplementary material for: Development and Validation of a Rapid High-Performance Liquid Chromatography Method for Simultaneous Determination of Methylxanthines and Flavanols in Cocoa Husk Tea
Source: Molecules. 2026 May 17;31(10):1697. doi: 10.3390/molecules31101697 (PMC13209721; doi:10.3390/molecules31101697)
Supplement: Supplementary file 1 [file molecules-31-01697-s001.zip › Figures S7–S9 and Tables S1-S3. Chromatograms and signal-to-noise data for determine LOD.pdf]

Supplementary Materials

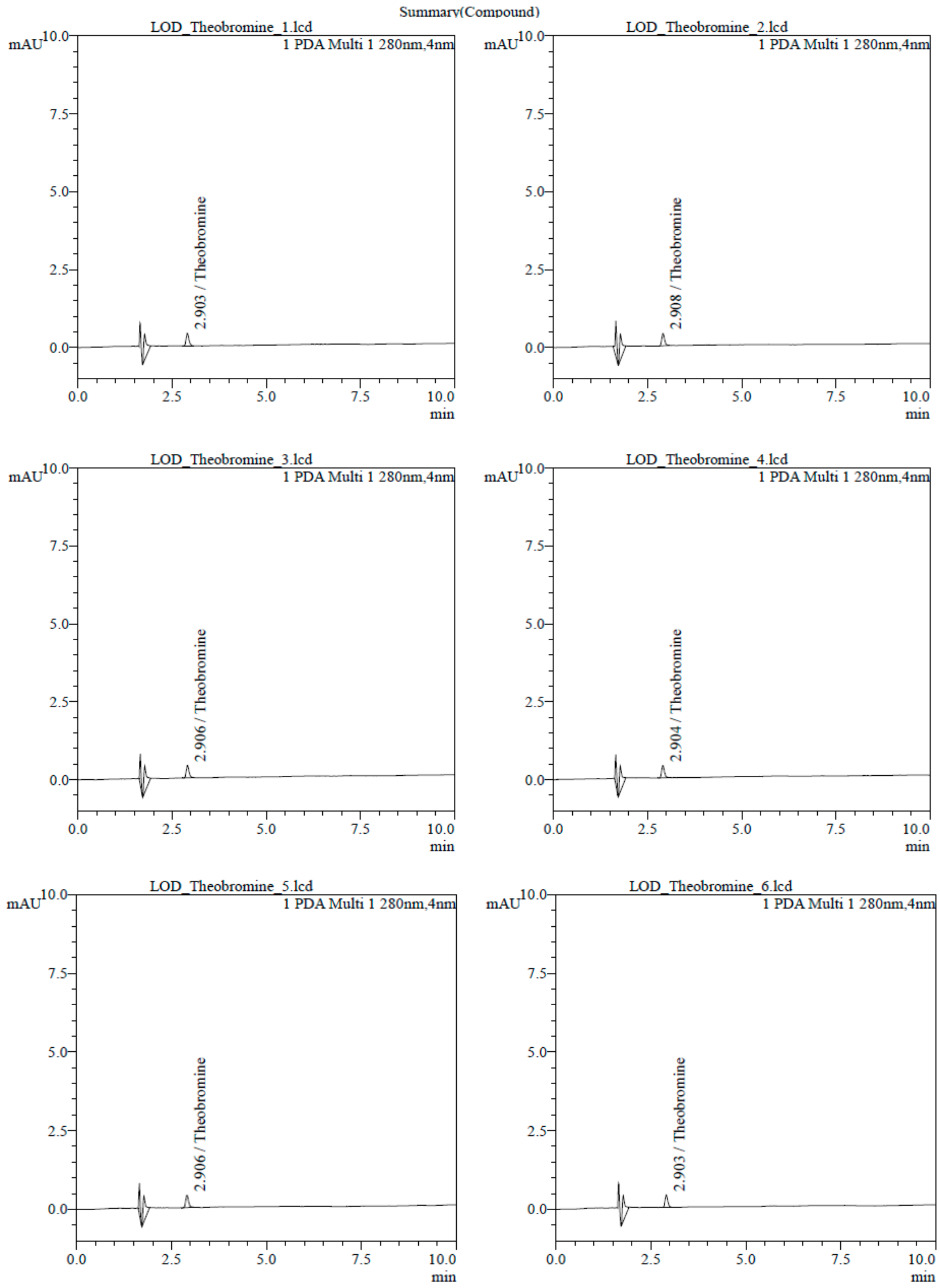

Figure S7. Chromatograms of Theobromine at the limit of detection (n=6)

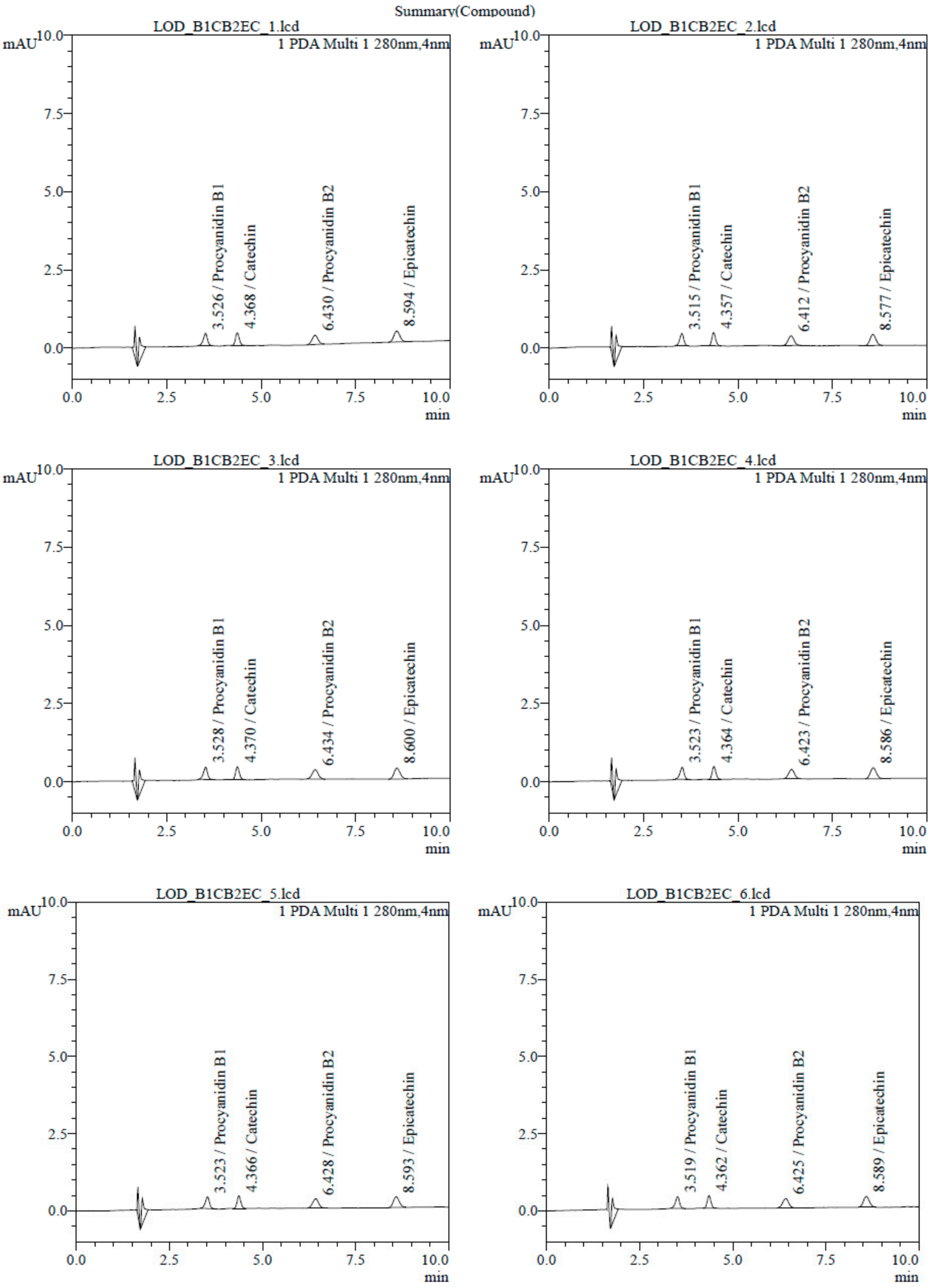

Figure S8. Chromatograms of Catechin, Epicatechin, Procyanidin B1, and Procyanidin B2 at the limit of detection (n=6)

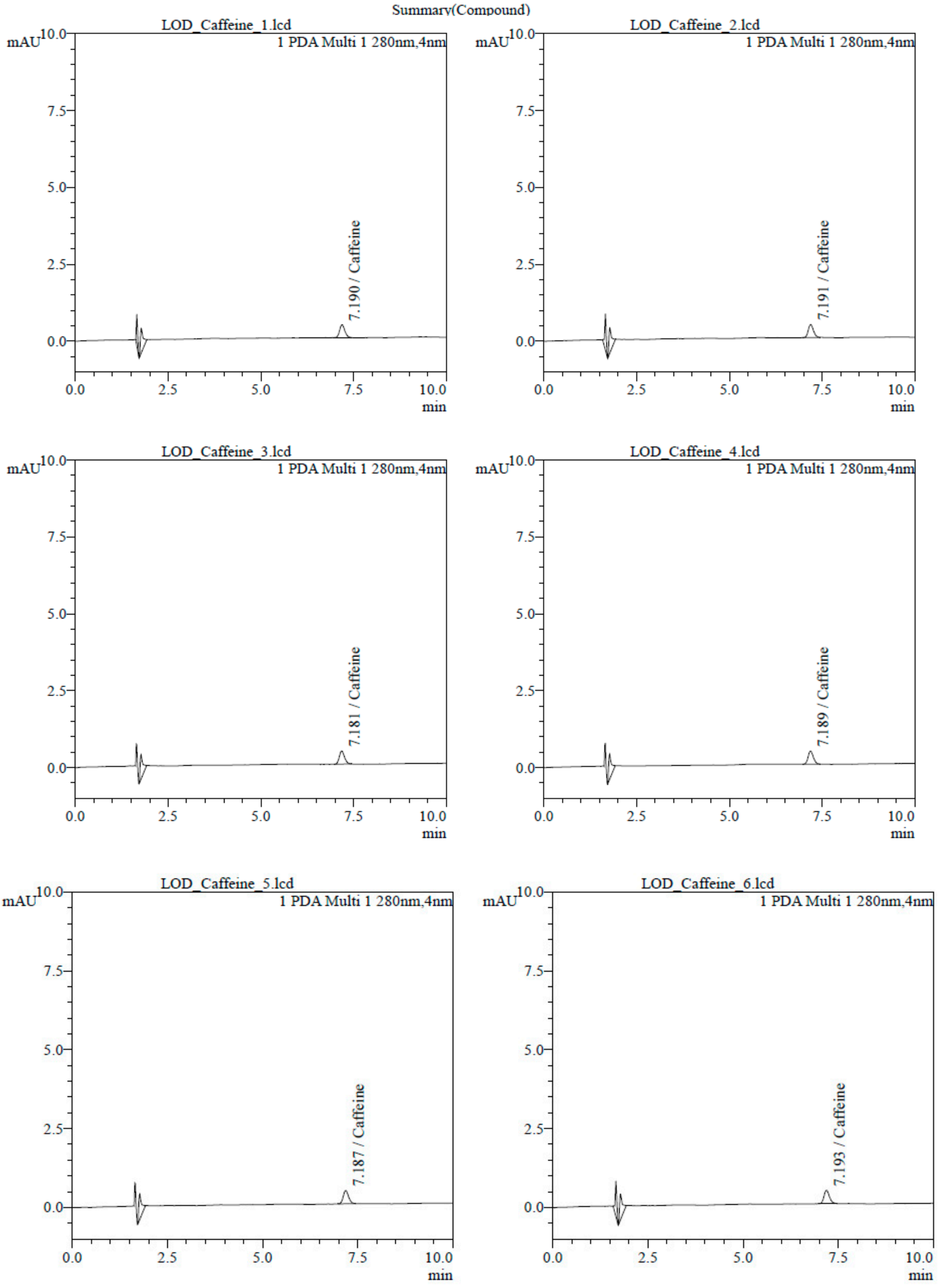

Figure S9. Chromatograms of Caffeine at the limit of detection (n=6)

Table S1. Signal-to-noise (S/N) ratios of Theobromine at 0.386 ppm (n=6)

Sample name	S/N
LOD_Theobromine_1	5.25
LOD_Theobromine_2	5.16
LOD_Theobromine_3	5.20
LOD_Theobromine_4	5.10
LOD_Theobromine_5	5.21
LOD_Theobromine_6	5.02
**Average**	**5.15**
**S/N ≥3**	**Pass**

Table S2. Signal-to-noise (S/N) ratios of Catechin, Epicatechin, Procyanidin B1, and Procyanidin B2 at 3.125 ppm (n=6)

Sample name	S/N	Average	S/N ≥3
LOD_Catechin_1	7.84	**4.98**	**Pass**
LOD_Catechin_2	4.69		
LOD_Catechin_3	4.33		
LOD_Catechin_4	4.24		
LOD_Catechin_5	4.32		
LOD_Catechin_6	4.46		
LOD_Epicatechin_1	6.74	**4.26**	**Pass**
LOD_ Epicatechin _2	3.96		
LOD_ Epicatechin _3	3.69		
LOD_ Epicatechin _4	3.62		
LOD_ Epicatechin _5	3.71		
LOD_ Epicatechin _6	3.85		
LOD_ Procyanidin B1 _1	7.48	**4.71**	**Pass**
LOD_ Procyanidin B1 _2	4.41		
LOD_ Procyanidin B1 _3	4.12		
LOD_ Procyanidin B1 _4	3.98		
LOD_ Procyanidin B1 _5	4.06		
LOD_ Procyanidin B1 _6	4.19		
LOD_ Procyanidin B2 _1	5.72	**3.64**	**Pass**
LOD_ Procyanidin B2 _2	3.46		
LOD_ Procyanidin B2 _3	3.16		
LOD_ Procyanidin B2 _4	3.06		
LOD_ Procyanidin B2 _5	3.13		
LOD_ Procyanidin B2 _6	3.29		

Table S3. Signal-to-noise (S/N) ratios of Caffeine at 0.800 ppm (n=6)

**Sample name**	**S/N**
LOD_ Caffeine _1	5.50
LOD_ Caffeine _2	5.50
LOD_ Caffeine _3	5.66
LOD_ Caffeine_4	5.62
LOD_ Caffeine _5	5.55
LOD_ Caffeine _6	5.62
**Average**	**5.57**
**S/N ≥3**	**Pass**
