# Supplementary material for: Development and Validation of a Rapid High-Performance Liquid Chromatography Method for Simultaneous Determination of Methylxanthines and Flavanols in Cocoa Husk Tea
Source: Molecules. 2026 May 17;31(10):1697. doi: 10.3390/molecules31101697 (PMC13209721; doi:10.3390/molecules31101697)
Supplement: Supplementary file 1 [file molecules-31-01697-s001.zip › Table S26. Acceptance criteria for precision and Tables S27-S31. Intraday precision in cocoa husk tea.pdf]

Supplementary Materials

Table S26. Expected precision (repeatability) criteria expressed as RSD_r ,% across different analyte concentrations.

Table A4. Expected precision (repeatability) as a function of analyte concentration[a]

Analyte, %	Mass fraction (C)	Unit	RSD_r, %
100	1	100%	1.3
10	10^{-1}	10%	1.9
1	10^{-2}	1%	2.7
0.1	10^{-3}	0.1%	3.7
0.01	10^{-4}	100 ppm (mg/kg)	5.3
0.001	10^{-5}	10 ppm (mg/kg)	7.3
0.0001	10^{-6}	1 ppm (mg/kg)	11
0.00001	10^{-7}	100 ppb (μg/kg)	15
0.000001	10^{-8}	10 ppb (μg/kg)	21
0.0000001	10^{-9}	1 ppb (μg/kg)	30

[a] Table excerpted from *AOAC Peer-Verified Methods Program, Manual on Policies and Procedures* (1998) AOAC INTERNATIONAL, Rockville, MD, USA.

The precision of a method is the closeness of agreement between independent test results obtained under stipulated conditions. Precision is usually expressed in terms of imprecision and computed as a relative standard deviation of the test results. The imprecision of a method increases as the concentration of the analyte decreases. This table provides targets RSDs for a range of analyte concentrations.

Table S27. Intra-day repeatability (RSD_r, %) of Theobromine determination in cocoa husk tea.

Repeat	Retention time	Conc. (mg g^{-1})
1	2.894	17.301
2	2.894	17.622
3	2.899	17.409
4	2.898	17.387
5	2.899	17.223
6	2.893	17.235
**Average**	**2.897**	**17.363**
**RSD_r ,%**	**0.091**	**0.852**
**RSD_r ,% Conc. (mg g^{-1}) ≤ 2.7**		**Pass**

Table S28. Intra-day repeatability (RSD_r, %) of Catechin determination in cocoa husk tea.

Repeat	Retention time	Conc. (mg g^{-1})
1	4.337	0.137
2	4.340	0.145
3	4.343	0.141
4	4.342	0.140
5	4.342	0.142
6	4.336	0.138
**Average**	**4.340**	**0.140**
**RSD_r ,%**	**0.066**	**1.978**
**RSD_r ,% Conc. (mg g^{-1}) ≤ 5.3**		**Pass**

Table S29. Intra-day repeatability (RSD_r, %) of Epicatechin determination in cocoa husk tea.

Repeat	Retention time	Conc. (mg g^{-1})
1	8.559	0.168
2	8.555	0.189
3	8.558	0.173
4	8.555	0.165
5	8.556	0.169
6	8.549	0.180
**Average**	**8.555**	**0.174**
**RSD_r,%**	**0.043**	**5.147**
**RSD_r,% Conc. (mg g^{-1}) ≤ 5.3**		**Pass**

Table S30. Intra-day repeatability (RSD_r, %) of Procyanidin B2 determination in cocoa husk tea.

Repeat	Retention time	Conc. (mg g^{-1})
1	6.333	0.317
2	6.352	0.334
3	6.341	0.311
4	6.342	0.311
5	6.340	0.308
6	6.337	0.323
**Average**	**6.341**	**0.317**
**RSD_r,%**	**0.099**	**3.137**
**RSD_r,% Conc. (mg g^{-1}) ≤ 5.3**		**Pass**

Table S31. Intra-day repeatability (RSD_r, %) of Caffeine determination in cocoa husk tea.

Repeat	Retention time	Conc. (mg g^{-1})
1	7.120	1.758
2	7.118	1.801
3	7.126	1.769
4	7.119	1.764
5	7.121	1.739
6	7.116	1.735
**Average**	**7.120**	**1.761**
**RSD_r,%**	**0.049**	**1.348**
**RSD_r,% Conc. (mg g^{-1}) ≤ 3.7**		**Pass**
