# Supplementary material for: Development and Validation of a Rapid High-Performance Liquid Chromatography Method for Simultaneous Determination of Methylxanthines and Flavanols in Cocoa Husk Tea
Source: Molecules. 2026 May 17;31(10):1697. doi: 10.3390/molecules31101697 (PMC13209721; doi:10.3390/molecules31101697)
Supplement: Supplementary file 1 [file molecules-31-01697-s001.zip › Table S7. Acceptance criteria for _recovery and Table S8-S25. _recovery from spiked at QL,QM and QH.pdf]

Supplementary Materials

Table S7. Acceptance criteria used for evaluating method accuracy, expressed as expected recovery ranges at different analyte concentrations.

Table A5. Expected recovery as a function of analyte concentration[a]

Analyte, %	Mass fraction (C)	Unit	Mean recovery, %
100	1	100%	98–102
10	10^{-1}	10%	
1	10^{-2}	1%	97–103
0.1	10^{-3}	0.1%	95–105
0.01	10^{-4}	100 ppm (mg/kg)	90–107
0.001	10^{-5}	10 ppm (mg/kg)	80–110
0.0001	10^{-6}	1 ppm (mg/kg)	
0.00001	10^{-7}	100 ppb (µg/kg)	
0.000001	10^{-8}	10 ppb (µg/kg)	60–115
0.0000001	10^{-9}	1 ppb (µg/kg)	40–120

[a] Table excerpted from *AOAC Peer-Verified Methods Program, Manual on Policies and Procedures* (1998) AOAC INTERNATIONAL, Rockville, MD, USA.

Recovery is defined as the ratio of the observed mean test result to the true value. The range of the acceptable mean recovery expands as the concentration of the analyte decreases. This table provides target mean recovery ranges for analyte concentrations from 100% to 1 ppb.

Table S8. Accuracy (% recovery) at the low-quality control level (QL) for Theobromine at a spiked at a concentration of 15.625 ppm.

Repeat	Area	Conc. ppm (mg/L)	% Recovery
1	94,629	13.216	84.58
2	94,453	13.186	84.39
3	94,562	13.205	84.51
4	94,591	13.210	84.54
5	94,474	13.190	84.41
6	94,705	13.229	84.67
**Average**	94,569	13.206	**84.52**
**% Recovery (80-110)**			**Pass**

Table S9. Accuracy (% recovery) at the low-quality control level (QL) for Catechin at a spiked at a concentration of 3.125 ppm.

Repeat	Area	Conc. ppm (mg/L)	% Recovery
1	5,110	2.578	82.49
2	5,098	2.566	82.11
3	5,079	2.547	81.50
4	5,116	2.584	82.69
5	5,121	2.589	82.85
6	5,094	2.562	81.98
**Average**	5,103	2.571	**82.27**
**% Recovery (80-110)**			**Pass**

Table S10. Accuracy (% recovery) at the low-quality control level (QL) for Epicatechin at a spiked at a concentration of 3.125 ppm.

Repeat	Area	Conc. ppm (mg/L)	% Recovery
1	4,760	2.782	89.02
2	4,810	2.818	90.19
3	4,823	2.828	90.50
4	4,966	2.934	93.89
5	4,780	2.796	89.48
6	4,702	2.738	87.63
**Average**	4,807	2.816	**90.12**
**% Recovery (80-110)**			**Pass**

Table S11. Accuracy (% recovery) at the low-quality control level (QL) for Procyanidin B1 at a spiked at a concentration of 3.125 ppm.

Repeat	Area	Conc. ppm (mg/L)	% Recovery
1	1,978	3.320	106.24
2	1,968	3.308	105.85
3	1,999	3.343	106.98
4	1,966	3.306	105.78
5	1,994	3.337	106.80
6	1,980	3.322	106.29
**Average**	1,981	3.323	**106.32**
**% Recovery (80-110)**			**Pass**

Table S12. Accuracy (% recovery) at the low-quality control level (QL) for Procyanidin B2 at a spiked at a concentration of 3.125 ppm.

Repeat	Area	Conc. ppm (mg/L)	% Recovery
1	7,010	3.411	109.15
2	7,010	3.410	109.12
3	6,840	3.216	102.91
4	6,976	3.371	107.87
5	6,880	3.262	104.38
6	7,026	3.430	109.76
**Average**	6,957	3.350	**107.20**
**% Recovery (80-110)**			**Pass**

Table S13. Accuracy (% recovery) at the low-quality control level (QL) for Caffeine at a spiked at a concentration of 9.375 ppm.

Repeat	Area	Conc. ppm (mg/L)	% Recovery
1	54,652	8.361	89.18
2	54,606	8.353	89.10
3	54,611	8.353	89.10
4	54,545	8.342	88.98
5	54,489	8.332	88.87
6	54,732	8.375	89.33
**Average**	54,606	8.352	**89.09**
**% Recovery (80-110)**			**Pass**

Table S14. Accuracy (% recovery) at the medium-quality control level (QM) for Theobromine at a spiked at a concentration of 125 ppm.

Repeat	Area	Conc. ppm (mg/L)	% Recovery
1	754,377	125.842	100.67
2	758,882	126.611	101.29
3	757,442	126.365	101.09
4	758,348	126.520	101.22
5	759,452	126.708	101.37
6	758,904	126.615	101.29
**Average**	757,901	126.443	**101.15**
**% Recovery (90-107)**			**Pass**

Table S15. Accuracy (% recovery) at the medium-quality control level (QM) for Catechin at a spiked at a concentration of 25 ppm.

Repeat	Area	Conc. ppm (mg/L)	% Recovery
1	26,879	24.403	97.61
2	28,421	25.949	103.79
3	28,407	25.934	103.74
4	28,339	25.867	103.47
5	27,545	25.071	100.28
6	27,620	25.146	100.58
**Average**	27,869	25.395	**101.58**
**% Recovery (80-110)**			**Pass**

Table S16. Accuracy (% recovery) at the medium-quality control level (QM) for Epicatechin at a spiked at a concentration of 25 ppm.

Repeat	Area	Conc. ppm (mg/L)	% Recovery
1	34,796	25.035	100.14
2	34,970	25.165	100.66
3	34,777	25.021	100.08
4	35,120	25.276	101.10
5	34,987	25.177	100.71
6	35,117	25.273	101.09
**Average**	34,961	25.158	**100.63**
**% Recovery (80-110)**			**Pass**

Table S17. Accuracy (% recovery) at the medium-quality control level (QM) for Procyanidin B1 at a spiked at a concentration of 25 ppm.

Repeat	Area	Conc. ppm (mg/L)	% Recovery
1	20,268	24.050	96.20
2	20,143	23.908	95.63
3	19,979	23.722	94.89
4	20,244	24.022	96.09
5	20,199	23.971	95.89
6	20,233	24.009	96.04
**Average**	20,178	23.947	**95.79**
**% Recovery (80-110)**			**Pass**

Table S18. Accuracy (% recovery) at the medium-quality control level (QM) for Procyanidin B2 at a spiked at a concentration of 25 ppm.

Repeat	Area	Conc. ppm (mg/L)	% Recovery
1	26,402	25.676	102.70
2	26,906	26.254	105.02
3	26,677	25.991	103.96
4	26,989	26.349	105.40
5	26,968	26.325	105.30
6	26,991	26.352	105.41
**Average**	26,822	26.158	**104.63**
**% Recovery (80-110)**			**Pass**

Table S19. Accuracy (% recovery) at the medium-quality control level (QM) for Caffeine at a spiked at a concentration of 75 ppm.

Repeat	Area	Conc. ppm (mg/L)	% Recovery
1	429,210	75.150	100.20
2	432,151	75.675	100.90
3	431,411	75.542	100.72
4	432,005	75.648	100.86
5	432,446	75.727	100.97
6	432,370	75.714	100.95
**Average**	431,599	75.576	**100.77**
**% Recovery (80-110)**			**Pass**

Table S20. Accuracy (% recovery) at the high-quality control level (QH) for Theobromine at a spiked at a concentration of 500 ppm.

Repeat	Area	Conc. ppm (mg/L)	% Recovery
1	2,950,537	500.748	100.15
2	2,939,964	498.943	99.79
3	2,940,986	499.118	99.82
4	2,943,013	499.463	99.89
5	2,945,721	499.926	99.99
6	2,940,435	499.023	99.80
**Average**	2,943,443	499.537	**99.91**
**% Recovery (90-107)**			**Pass**

Table S21. Accuracy (% recovery) at the high-quality control level (QH) for Catechin at a spiked at a concentration of 100 ppm.

Repeat	Area	Conc. ppm (mg/L)	% Recovery
1	102,179	99.896	99.90
2	101,828	99.544	99.54
3	101,661	99.377	99.38
4	102,052	99.769	99.77
5	101,937	99.654	99.65
6	102,009	99.726	99.73
**Average**	101,944	99.661	**99.66**
**% Recovery (90-107)**			**Pass**

Table S22. Accuracy (% recovery) at the high-quality control level (QH) for Epicatechin at a spiked at a concentration of 100 ppm.

Repeat	Area	Conc. ppm (mg/L)	% Recovery
1	135,987	100.010	100.01
2	135,465	99.623	99.62
3	135,584	99.712	99.71
4	135,641	99.754	99.75
5	135,795	99.868	99.87
6	135,454	99.616	99.62
**Average**	135,654	99.764	**99.76**
**% Recovery (90-107)**			**Pass**

Table S23. Accuracy (% recovery) at the high-quality control level (QH) for Procyanidin B1 at a spiked at a concentration of 100 ppm.

Repeat	Area	Conc. ppm (mg/L)	% Recovery
1	87,721	100.503	100.50
2	87,296	100.021	100.02
3	87,220	99.935	99.93
4	87,405	100.144	100.14
5	87,457	100.204	100.20
6	87,225	99.940	99.94
**Average**	87,387	100.124	**100.12**
**% Recovery (90-107)**			**Pass**

Table S24. Accuracy (% recovery) at the high-quality control level (QH) for Procyanidin B2 at a spiked at a concentration of 100 ppm.

Repeat	Area	Conc. ppm (mg/L)	% Recovery
1	90,394	99.146	99.15
2	89,934	98.617	98.62
3	90,112	98.823	98.82
4	90,193	98.915	98.92
5	90,339	99.083	99.08
6	90,086	98.793	98.79
**Average**	90,176	98.896	**98.90**
**% Recovery (90-107)**			**Pass**

Table S25. Accuracy (% recovery) at the high-quality control level (QH) for Caffeine at a spiked at a concentration of 300 ppm.

Repeat	Area	Conc. ppm (mg/L)	% Recovery
1	1,694,465	300.764	100.25
2	1,686,406	299.327	99.78
3	1,688,902	299.772	99.92
4	1,689,703	299.915	99.97
5	1,690,728	300.098	100.03
6	1,688,678	299.732	99.91
**Average**	1,689,814	299.935	**99.98**
**% Recovery (90-107)**			**Pass**
