# Supplementary material for: Development and Validation of a Rapid High-Performance Liquid Chromatography Method for Simultaneous Determination of Methylxanthines and Flavanols in Cocoa Husk Tea
Source: Molecules. 2026 May 17;31(10):1697. doi: 10.3390/molecules31101697 (PMC13209721; doi:10.3390/molecules31101697)
Supplement: Supplementary file 1 [file molecules-31-01697-s001.zip › Tables 32-36 Interday precision in cocoa husk tea.pdf]

Supplementary Materials

Table S32. Inter-day repeatability (3 days) of Theobromine determination in cocoa husk tea.

Sample name	Retention time	Conc. (mg g^{-1})
Day1_1	2.894	17.301
Day1_2	2.894	17.622
Day1_3	2.899	17.409
Day1_4	2.898	17.387
Day1_5	2.899	17.223
Day1_6	2.893	17.235
Day2_1	2.905	17.400
Day2_2	2.906	17.545
Day2_3	2.909	17.390
Day2_4	2.902	17.503
Day2_5	2.899	17.435
Day2_6	2.901	17.200
Day3_1	2.917	17.537
Day3_2	2.920	17.361
Day3_3	2.920	17.411
Day3_4	2.917	17.249
Day3_5	2.917	17.166
Day3_6	2.913	17.110
**Average**	**2.906**	**17.360**
**RSD_r,%**	**0.322**	**0.820**
**RSD_r,% Conc. (mg g^{-1}) ≤ 2.7**		**Pass**

Table S33. Inter-day repeatability (3 days) of Catechin determination in cocoa husk tea.

Sample name	Retention time	Conc. (mg g^{-1})
Day1_1	4.337	0.137
Day1_2	4.340	0.145
Day1_3	4.343	0.141
Day1_4	4.342	0.140
Day1_5	4.342	0.142
Day1_6	4.336	0.138
Day2_1	4.344	0.133
Day2_2	4.347	0.132
Day2_3	4.349	0.131
Day2_4	4.343	0.136
Day2_5	4.339	0.132
Day2_6	4.341	0.135
Day3_1	4.364	0.125
Day3_2	4.369	0.129
Day3_3	4.369	0.130
Day3_4	4.365	0.122
Day3_5	4.361	0.127

Day3_6	4.360	0.127
**Average**	**4.350**	**0.133**
**RSD_r,%**	**0.267**	**4.769**
**RSD_r,% Conc. (mg g^{-1}) ≤ 5.3**		**Pass**

Table S34. Inter-day repeatability (3 days) of Epicatechin determination in cocoa husk tea.

Sample name	Retention time	Conc. (mg g^{-1})
Day1_1	8.559	0.168
Day1_2	8.555	0.189
Day1_3	8.558	0.173
Day1_4	8.555	0.165
Day1_5	8.556	0.169
Day1_6	8.549	0.180
Day2_1	8.558	0.166
Day2_2	8.564	0.167
Day2_3	8.559	0.171
Day2_4	8.552	0.173
Day2_5	8.555	0.180
Day2_6	8.553	0.155
Day3_1	8.618	0.170
Day3_2	8.642	0.178
Day3_3	8.619	0.174
Day3_4	8.643	0.183
Day3_5	8.613	0.175
Day3_6	8.625	0.170
**Average**	**8.580**	**0.173**
**RSD_r,%**	**0.408**	**4.465**
**RSD_r,% Conc. (mg g^{-1}) ≤ 5.3**		**Pass**

Table S35. Inter-day repeatability (3 days) of Procyanidin B2 determination in cocoa husk tea.

Sample name	Retention time	Conc. (mg g^{-1})
Day1_1	6.333	0.317
Day1_2	6.352	0.334
Day1_3	6.341	0.311
Day1_4	6.342	0.311
Day1_5	6.340	0.308
Day1_6	6.337	0.323
Day2_1	6.345	0.312
Day2_2	6.344	0.317
Day2_3	6.358	0.324
Day2_4	6.345	0.322
Day2_5	6.349	0.331
Day2_6	6.330	0.306
Day3_1	6.338	0.306
Day3_2	6.336	0.310

Day3_3	6.337	0.305
Day3_4	6.328	0.298
Day3_5	6.334	0.306
Day3_6	6.327	0.315
**Average**	**6.340**	**0.314**
**RSD_r,%**	**0.131**	**3.037**
**RSD_r,% Conc. (mg g^{-1}) ≤ 5.3**		**Pass**

Table S36. Inter-day repeatability (3 days) of Caffeine determination in cocoa husk tea.

Sample name	Retention time	Conc. (mg g^{-1})
Day1_1	7.120	1.758
Day1_2	7.118	1.801
Day1_3	7.126	1.769
Day1_4	7.119	1.764
Day1_5	7.121	1.739
Day1_6	7.116	1.735
Day2_1	7.148	1.774
Day2_2	7.152	1.796
Day2_3	7.151	1.780
Day2_4	7.142	1.788
Day2_5	7.143	1.777
Day2_6	7.142	1.744
Day3_1	7.202	1.814
Day3_2	7.221	1.804
Day3_3	7.210	1.805
Day3_4	7.223	1.787
Day3_5	7.200	1.768
Day3_6	7.207	1.762
**Average**	**7.159**	**1.776**
**RSD_r,%**	**0.551**	**1.313**
**RSD_r,% Conc. (mg g^{-1}) ≤ 3.7**		**Pass**
